# Safety and efficacy of ex vivo expanded CD34^+^ stem cells in murine and primate models

**DOI:** 10.1186/s13287-019-1275-0

**Published:** 2019-06-13

**Authors:** Yu Zhang, Bin Shen, Xin Guan, Meng Qin, Zhihua Ren, Yupo Ma, Wei Dai, Xinxin Ding, Yongping Jiang

**Affiliations:** 1 Biopharmaceutical R&D Center, Chinese Academy of Medical Sciences & Peking Union Medical College, Suzhou, 215126 China; 2 Biopharmagen Corp, Suzhou, 215126 China; 3 Beijing Advanced Innovation Center for Soft Matter Science and Engineering, College of Life Science and Technology, Beijing University of Chemical Technology, Beijing, 100029 China; 4 Department of Pathology, BST-9C, The State University of New York at Stony Brook, Stony Brook, NY 11794 USA; 5 Department of Environmental Medicine, NYU Langone Medical Center, Tuxedo, NY 10987 USA; 6 Department of Pharmacology and Toxicology, College of Pharmacy, University of Arizona, Tucson, AZ 85721 USA

**Keywords:** Long-term HSC, Ex vivo expansion, Human cord blood, Transplantation, NOD/SCID mice, Nonhuman primates

## Abstract

**Background:**

Hematopoietic stem cell (HSC) transplantation has been widely applied to the treatment of malignant blood diseases. However, limited number of functional HSCs hinders successful transplantation. The purpose of our current study is to develop a new and cost-efficient medium formulation that could greatly enhance the expansion of HSCs while retaining their long-term repopulation and hematopoietic properties for effective clinical transplantation.

**Methods:**

Enriched human CD34^+^ cells and mobilized nonhuman primate peripheral blood CD34^+^ cells were expanded with a new, cost-efficient expansion medium formulation, named hematopoietic expansion medium (HEM), consisting of various cytokines and nutritional supplements. The long-term repopulation potential and hematologic-lineage differentiation ability of expanded human cells were studied in the non-obese diabetic/severe combined immunodeficiency mouse model. Furthermore, the efficacy and safety studies were performed by autologous transplantation of expanded primate cells in the nonhuman primate model.

**Results:**

HEM could effectively expand human CD34^+^ cells by up to 129 fold within 9 days. Expanded HSCs retained long-term repopulation potential and hematologic-lineage differentiation ability, as indicated by (1) maintenance (over unexpanded HSCs) of immunophenotypes of CD38^−^CD90^+^CD45RA^−^CD49f^+^ in CD34^+^ cells after expansion; (2) significant presence of multiple human hematopoietic lineages in mouse peripheral blood and bone marrow following primary transplantation; (3) enrichment (over unexpanded HSCs) in SCID-repopulating cell frequency measured by limiting dilution analysis; and (4) preservation of both myeloid and lymphoid potential among human leukocytes from mouse bone marrow in week 24 after primary transplantation or secondary transplantation. Moreover, the results of autologous transplantation in nonhuman primates demonstrated that HEM-expanded CD34^+^ cells could enhance hematological recovery after myelo-suppression. All primates transplanted with the expanded autologous CD34^+^ cells survived for over 18 months without any noticeable abnormalities.

**Conclusions:**

Together, these findings demonstrate promising potential for the utility of HEM to improve expansion of HSCs for clinical application.

**Electronic supplementary material:**

The online version of this article (10.1186/s13287-019-1275-0) contains supplementary material, which is available to authorized users.

## Background

Hematopoietic stem cells (HSCs) are capable of self-renewal and differentiation into all types of blood cells, including those of the erythroid, myeloid, and lymphoid lineages. HSCs are used as cellular therapeutic agents in the clinic for treating a variety of hematopoietic malignancies, including leukemia [1–4].

At present, three different sources of HSCs are used for transplantation, including umbilical cord blood (UCB), mobilized peripheral blood (mPB), and bone marrow (BM) [5, 6]. Due to limited availability of these cells for clinical transplantation, many attempts at ex vivo expansion have been made in the past to obtain a sufficient number of transplantable HSCs [7–9]. A vast majority of strategies for expanding HSCs ex vivo are focused on regulating the renewal and survival of HSCs mediated by intrinsic factors (e.g., transcription factors and signaling molecules) or environmental cues (e.g., cytokines, chemokines, stromal cells, and adhesion molecules) [10, 11]. Reasonable stem cell expansion has been achieved by co-culturing with BM mesenchymal stromal cells, with immortalized stromal cells [12–14] or by over-expression of self-renewal genes such as *HOXB4* [15–17] and Sall4b [18, 19]. However, these approaches involve the manipulation of the stromal environment or perturbation of the HSC genome, at the risk of unintended adverse effects, such as carcinogenesis. Therefore, it is imperative to develop a simple but effective approach for HSC expansion. Over the past two decades, various cytokines, small molecules, and chemicals have been used to expand HSCs ex vivo, including thrombopoietin (TPO) [20], Fms-related tyrosine kinase 3 ligand (Flt-3 L) [21], granulocyte colony-stimulating factor (G-CSF) [22], interleukin (IL)-3, -6, -11 [23, 24], stem cell factor (SCF) [25–27], angiopoietin (Ang)-1 [28], StemRegenin 1 (SR1) [29], and tetraethylenpentamin (TEPA) [30]. However, only limited success has been achieved with stem cell expansion by using a mixture of growth factors/cytokines/chemicals. In addition, the high cost of culturing HSCs ex vivo is another barrier for industrialization and clinical use. Thus, there is still a large need to further optimize efficiency and decrease cost.

In addition, most in vivo studies in the past have been related to engraftment in murine models, which is limited by their relatively short life span and the species differences between humans and mice. Conversely, the overwhelming homology between humans and primates allows for many of the same cytokines, growth factors, drugs, and therapeutic regimens to be used, and the regulation of hematopoiesis in primates is also physiologically similar to humans [31, 32]. Thus, nonhuman primates are considered to be a valuable mammalian model for cell transplantation studies.

In the present study, we developed a new and cost-efficient CD34^+^ HSC expansion medium formulation (denoted as HEM), which could effectively expand HSCs while retaining their long-term repopulation and hematopoietic ability on a large scale within 9 days. We also verified the long-term engraftment and repopulating ability of the expanded cells in the non-obese diabetic/severe combined immunodeficiency (NOD/SCID) mouse model and assessed their safety and efficacy in the nonhuman primate model.

## Methods

### Animals

All experiments that involved the use of animals were strictly conducted according to the standard guidelines. Female NOD/SCID mice (specific pathogen-free; 6 weeks old, weight 16.0–17.6 g), obtained from the Experimental Animal Center of Soochow University (Suzhou, China), were used for human UCB HSCs transplantation. The experimental protocols used for mice were approved by the Institutional Animal Care and Use Committees of Soochow University (IACUC permit number: SYXK (Su) 2013-0064).

Cynomolgus primates were obtained from the Medical Primate Research Center of the Institute of Medical Biology, Chinese Academy of Medical Sciences, and were housed and bred according to the Guidelines of the Experimental Animals Ethics Committee at the Institute of Medical Biology of the Chinese Academy of Medical Sciences (Permit Number YISHENGLUNZI [2014] 07), which complied with the humane regulations of replacement, refinement, and reduction (the 3 Rs).

### Isolation of CD34^+^ cells

Fresh UCB samples (within 6–8 h of collection) and cryopreserved specimens from anonymous donors were provided by the Nanjing Medical University Affiliated Suzhou Municipal Hospital (Suzhou, China). The study was approved by the Hospital’s Ethics Committee and Research Ethics Advisory Committee. CD34^+^ cells were enriched from mononuclear cells (MNCs) using the magnetic-activated cell sorting (MACS) immunomagnetic absorption column separation device and the CD34 MicroBead kit according to the manufacturer’s instructions (Miltenyi Biotec, Bergisch Gladbach, Germany). MNCs were obtained by density gradient centrifugation using Ficolle-Hypaque Premium (GE healthcare, NJ, USA). The purity of isolated CD34^+^ cells ranged from 90 to 99% as determined by flow cytometry using an anti-human CD34 mAb conjugated with phycoerythrin (PE; BD Biosciences, NJ, USA).

CD34^+^ stem cells from cynomolgus primates (*n* = 11) were mobilized with recombinant human (rh) G-CSF (200 μg/kg) and rhSCF (200 μg/kg) administered s.c. for five successive days. PB samples (15–20 mL each) were then collected at 24 and 48 h after the final injection. CD34^+^ cell enrichment was achieved using anti-CD34 IgM and MACS IgM microbeads (Miltenyi Biotec, Bergisch Gladbach, Germany) according to the manufacturer’s instructions and as described previously [33, 34]. The average purity of CD34^+^ cells in the enriched product was about 69.6% (ranging from 61.5 to 76%) as determined by flow cytometry. CD34^+^ and CD34^−^ cells were also cryopreserved separately and were revived at the time of autologous transplantation.

### Condition optimization for human HSC expansion

CD34^+^ cells derived from human UCB were cultured in Iscove’s modified Dulbecco’s medium (IMDM) with added nutrition supplements [25, 27, 35, 36], which consisted of putrescine (100 μM), selenium (5 ng/mL), insulin (25 μg/mL), transferrin (50 μg/mL), human serum albumin (1%), and B-27 Supplement (Life Technologies, NY, USA). Then, different combinations and concentrations of growth factors were added to figure out the optimal conditions, which could best support human CD34^+^ cell expansion ex vivo (Table 1). Agents tested included SCF (200 ng/mL), Flt-3 L (200 ng/mL), TPO (range 0–100 ng/mL), interleukin-3 (IL-3, range 0–100 ng/mL), G-CSF (range 0–100 ng/mL), GM-CSF (range 0–100 ng/mL), and SR1 (0.5–1 μM; Selleck Chemicals, USA). The final optimized expansion medium was named hematopoietic expansion medium (HEM). All cytokines were purchased from Biopharmagen Corp., Suzhou, China, unless otherwise specified. All the cells were kept and diluted with fresh medium everyday as required to maintain proper cell density ranging from 2 × 10^5^ to 5 × 10^5^ cells/mL in T25, T75 flasks and turning-bottle device (Corning, USA).

**Table 1:** Culture formula optimization for human CD34^+^ cell expansion

Group	IMDM	Nutrition Supplements	SCF (ng/mL)	Flt-3 L (ng/mL)	TPO (ng/mL)	IL-3 (ng/mL)	G-CSF (ng/mL)	GM-CSF (ng/mL)	SR1 (μM)
GF0	+	–	–	–	–	–	–	–	–
SGF0	+	+	–	–	–	–	–	–	–
SGF2	+	+	200	200	0	–	–	–	–
SGF3	+	+	200	200	5	–	–	–	–
	+	+			10	–	–	–	–
	+	+			20	–	–	–	–
	+	+			50	–	–	–	–
	+	+			100	–	–	–	–
SGF4	+	+	200	200	20	5	–	–	–
	+	+				10	–	–	–
	+	+				15	–	–	–
	+	+				25	–	–	–
	+	+				50	–	–	–
	+	+				100	–	–	–
SGF5	+	+	200	200	20	15	5	–	–
	+	+					10	–	–
	+	+					15	–	–
	+	+					25	–	–
	+	+					50	–	–
	+	+					100	–	–
SGF6	+	+	200	200	20	15	10	5	–
	+	+						10	–
	+	+						15	–
	+	+						25	–
	+	+						50	–
	+	+						100	–
SGF7	+	+	200	200	20	15	10	10	0.5
	+	+							1
	+	+							2
HEM	+	+	200	200	20	15	10	10	–

The composition of the base (GF0 and SGF0), intermediate (SGF2-SGF7), and the final optimized media formula (HEM) are shown

### Labeling of primate mPB-derived expanded cells

Green fluorescent protein (GFP) lentivirus was prepared as previously described [37]. Primate CD34^+^ cells were cultured in the HEM. After 6-day expansion, cells were transduced with GFP lentivirus for 8 h and then expanded for three additional days. Cells were subsequently harvested for further analyses and transplantation. The transduction efficiency of GFP lentiviral particles was confirmed by flow cytometry.

### Flow cytometry

Flow cytometry data were collected using a FACSVerse® system (Becton Dickinson, NJ, USA) and analyzed using FlowJo® software. At least 10,000 events were acquired for each sample. The samples were analyzed for expression of cell surface markers using fluorescein isothiocyanate (FITC)-labeled antibodies against CD38, CD15, CD66, and CD42b; PE-labeled antibodies against CD14, CD34, CD49f, and CD45; PE-CY5-labeled antibody against CD90; Allophycocyanin (APC)-H7-labeled antibody against CD45RA; and APC-labeled antibodies against CD34, CD19, CD71, and CD41a. Fluorescein isothiocyanate, PE, PE-CY5, APC-H7, or APC-conjugated isotype-matched preimmune antibodies served as controls. All antibodies were purchased from Becton Dickinson.

### Cell counting and growth curve assays

Cells were counted at various time points using the Count Star® cell counting system. The number of CD34^+^ cells in each group was calculated based on the number of total nucleated cells and the percent of CD34^+^ cells as determined by flow cytometric analysis.

### Colony forming unit assays

MethoCult H4230 methylcellulose medium (Stem Cell Technologies, Vancouver, Canada) was thawed overnight at 4 °C. Unexpanded CD34^+^ cells and 9-day HEM-expanded CD34^+^ cells were prepared at the required final plating concentration of 1 × 10^4^ cells per dish. Duplicate cultures were prepared with 1.1 mL cell suspension in each 35-mm dish. The cells were incubated at 37 °C in 5% CO_2_ with > 95% humidity for approximately 16 days. Various colony-forming units (CFUs), including erythroid burst-forming unit (BFU-E), granulocyte-monocyte colony-forming unit (CFU-GM), megakaryocyte colony-forming unit (CFU-M), and granulocyte-erythrocyte-macrophage-megakaryocyte colony-forming unit (CFU-GEMM) were counted with a bright-field microscope. A colony with > 100 cells was counted as a positive colony.

### Transplantation of expanded human HSCs in NOD/SCID mice

In vivo NOD/SCID transplantation studies were conducted using human UCB CD34^+^ cells. A total of 64 mice were transplanted in this study. Sixteen mice were randomly assigned to each of the following treatment groups: normal saline; no expansion (transplanted with 1 × 10^5^ unexpanded CD34^+^ cells per mouse); expansion with HEM (low dose, transplanted with 1 × 10^5^ expanded CD34^+^ cells per mouse); and expansion with HEM (high dose, transplanted with CD34^+^ cells expanded for 9 days from 1 × 10^5^ starting CD34^+^ cells). Cells were intravenously infused into NOD/SCID mice that were pretreated with 2.5 Gy ^60^Co, a sub-lethal dose of radiation. Mouse PB samples were collected from the retro-orbital plexus at different time points after transplantation and were stained with various antibodies as indicated in the Flow cytometry section.

Long-term engraftment was further determined by secondary transplantation in NOD/SCID mice (*n* = 8). BM cells harvested from both femurs/tibias were resuspended in saline and then injected into sublethally irradiated (2.5 Gy, ^60^Co) secondary NOD/SCID recipients at a ratio of one donor to one recipient.

### Limiting dilution assays

The frequency of SCID-repopulating cell (SRC) in the unexpanded or HEM-expanded cell population was determined by injecting cohorts of mice (*n* = 40) with several dilutions of cells (CD34^+^ cell number at 5000, 10000, 20,000, 30,000). Human CD45^+^ cell proportions in mouse BM were determined by flow cytometry in week 8 post cell injection. Mice with human CD45^+^ cell ratio lower than 0.5% were defined as a negative. The SRC frequency was calculated from the proportions of negative mice in each cohort, using the L-CALC™ software (STEMCELL™ Technologies, Vancouver, BC, Canada), which employed Poisson statistics and the method of maximum likelihood. After log transformation, the ratio of frequencies was computed as reported earlier [38–40].

### CD34^+^ cell autologous transplantation in cynomolgus primates

Cynomolgus primates were used to examine the ability to reconstruct hematopoietic lineage of HEM-expanded primate CD34^+^ cells. Transplantation was performed at least 1 month after BM mobilization and collection. The primates (*n* = 11) were given cyclophosphamide (CTX; Energy Chemical, Shanghai, China) by intravenous injection (i.v.) at a dose of 50 mg/kg/day for successively 2 days. Nonhuman primates were randomly divided into following three groups: control group (*n* = 3) treated with normal saline, negative control group (*n* = 3) transplanted with autologous CD34^−^ cells, and the experimental group (*n* = 5) transplanted with GFP-labeled, HEM-expanded CD34^+^ cells along with CD34^−^ cells. Transplantation was conducted on day 3 after the last CTX injection when the animals exhibited apparent myelo-suppression.

After transplantation, routine blood tests were performed using the Sysmex XT-2000iv system (Sysmex corporation, KoBE, Japan) to monitor hematopoietic cell recovery including white blood cells (WBC), neutrophils (NEU), lymphocytes (LYM), and platelets (PLT). PB cells were stained for CD45, CD14, and CD20, and then analyzed by flow cytometry to determine the contribution of GFP^+^ cells to different subsets. One month after transplantation, GFP^+^ cells in the CD45^+^ cell population derived from primate BM was also determined.

### Statistical analysis

One-way analysis of variance (ANOVA) followed by Dunnett’s multiple comparison test was used for comparisons among the various treatment groups. Student’s *t* test was used for comparisons between two groups. Results were considered statistically significant when the *p* value was less than 0.05.

## Results

### Growth factor optimization for human HSC expansion

**Fig. 1:**
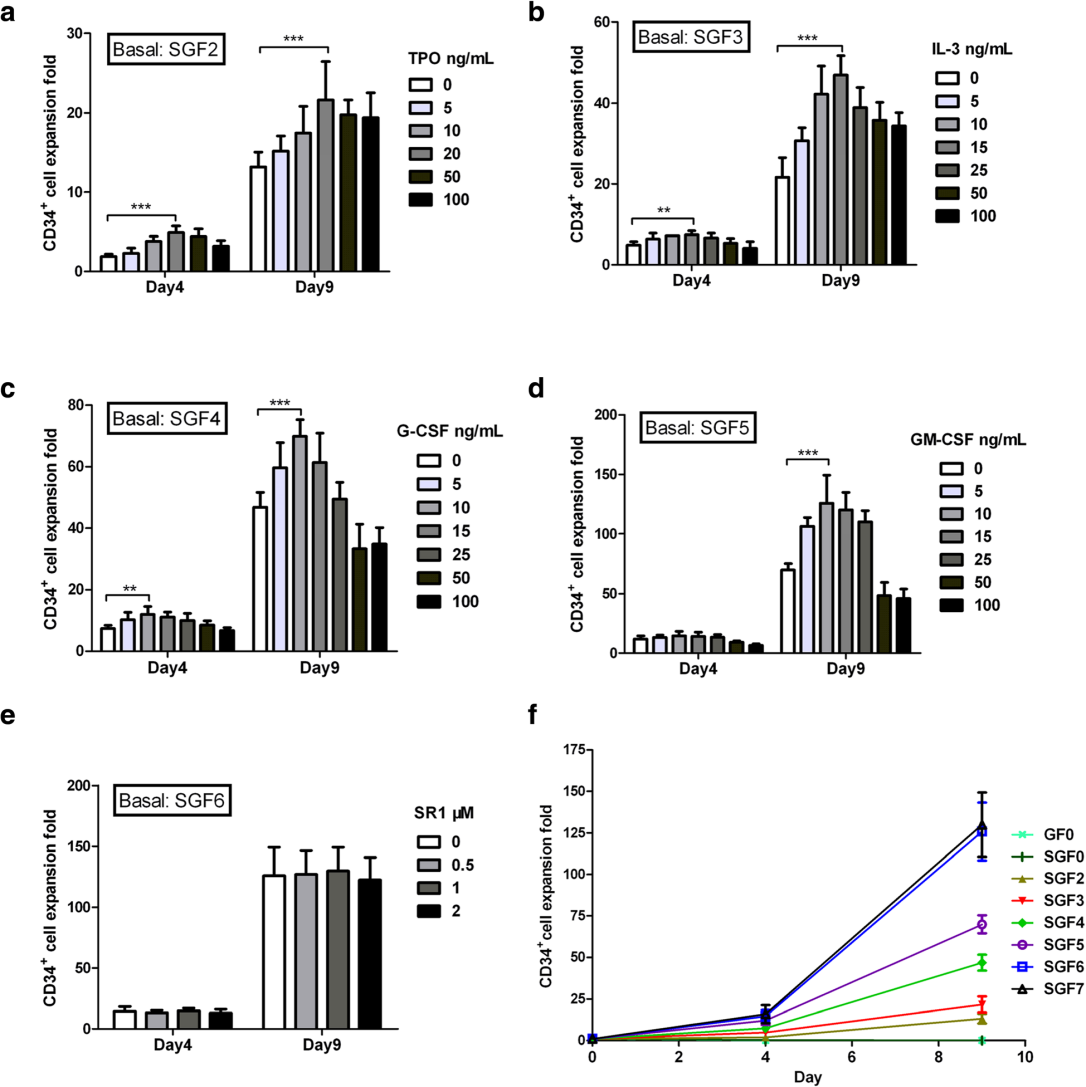
Effect of different medium conditions on HSC expansion ex vivo. **a** CD34^+^ cell expansion with TPO concentrations ranging from 0 to 100 ng/mL in SGF2 medium (IMDM supplemented with 200 ng/mL SCF, 200 ng/mL Flt-3 L, and nutrition supplements). **b** CD34^+^ cell expansion with IL-3 concentrations ranging from 0 to 100 ng/mL in SGF3 medium (SGF2 with 20 ng/mL TPO). **c** CD34^+^ cell expansion with G-CSF concentrations ranging from 0 to 100 ng/mL in SGF4 medium (SGF3 with 15 ng/mL IL-3). **d** CD34^+^ cell expansion with GM-CSF concentrations ranging from 0 to 100 ng/mL in SGF5 medium (SGF4 with 10 ng/mL G-CSF). **e** CD34^+^ cell expansion with SR1 concentrations ranging from 0 to 2 μM in SGF6 medium (SGF5 with 10 ng/mL GM-CSF). **f** A summary of CD34^+^ cell expansion in media with different growth factor combinations. SGF7 is SGF6 supplemented with 1 μM SR1. Expansion was calculated as fold increase (after/before expansion) in cell counts on each day (days 0, 4, and 9). Data are shown as mean ± SD, *n* = 6. **p < 0.01, ***p < 0.001; one-way ANOVA followed by Dunnett’s multiple comparison test

Various growth factors reagents, including TPO, IL-3, G-CSF, GM-CSF, and SR1, were added one by one, or in combination, into the media IMDM supplemented with nutrition supplements, as well as SCF and Flt-3 L, to identify the optimal concentrations needed for expanding human HSCs ex vivo (Table 1). The expansion fold of CD34^+^ cell was found to be maximal with TPO at 20 ng/mL, IL-3 at 15 ng/mL, G-CSF at 10 ng/mL, and GM-CSF at 10 ng/mL. When higher concentrations of these growth factors were used, CD34^+^ cell number was reduced, because the CD34^+^ ratio was decreased (Fig. 1a–d). Our findings regarding the doses of TPO and G-CSF were consistent with the literature [36, 37]. On the other hand, SR1 did not elicit significant enhancement of CD34^+^ cell expansion when added to media supplemented with the six cytokines and nutrition supplements (Fig. 1e). Therefore, SR1 was excluded from subsequent experiments. At the optimized concentrations of each growth factor, the combination of the six cytokines showed the best capability in HSC expansion (Fig. 1f). The final optimized HEM for human HSC expansion was IMDM supplemented with six cytokines (SCF, TPO, Flt-3 L, IL-3, G-CSF, and GM-CSF) and nutrition supplements as indicated in Table 1.

### HEM promotes expansion of HSCs isolated from various sources

**Fig. 2:**
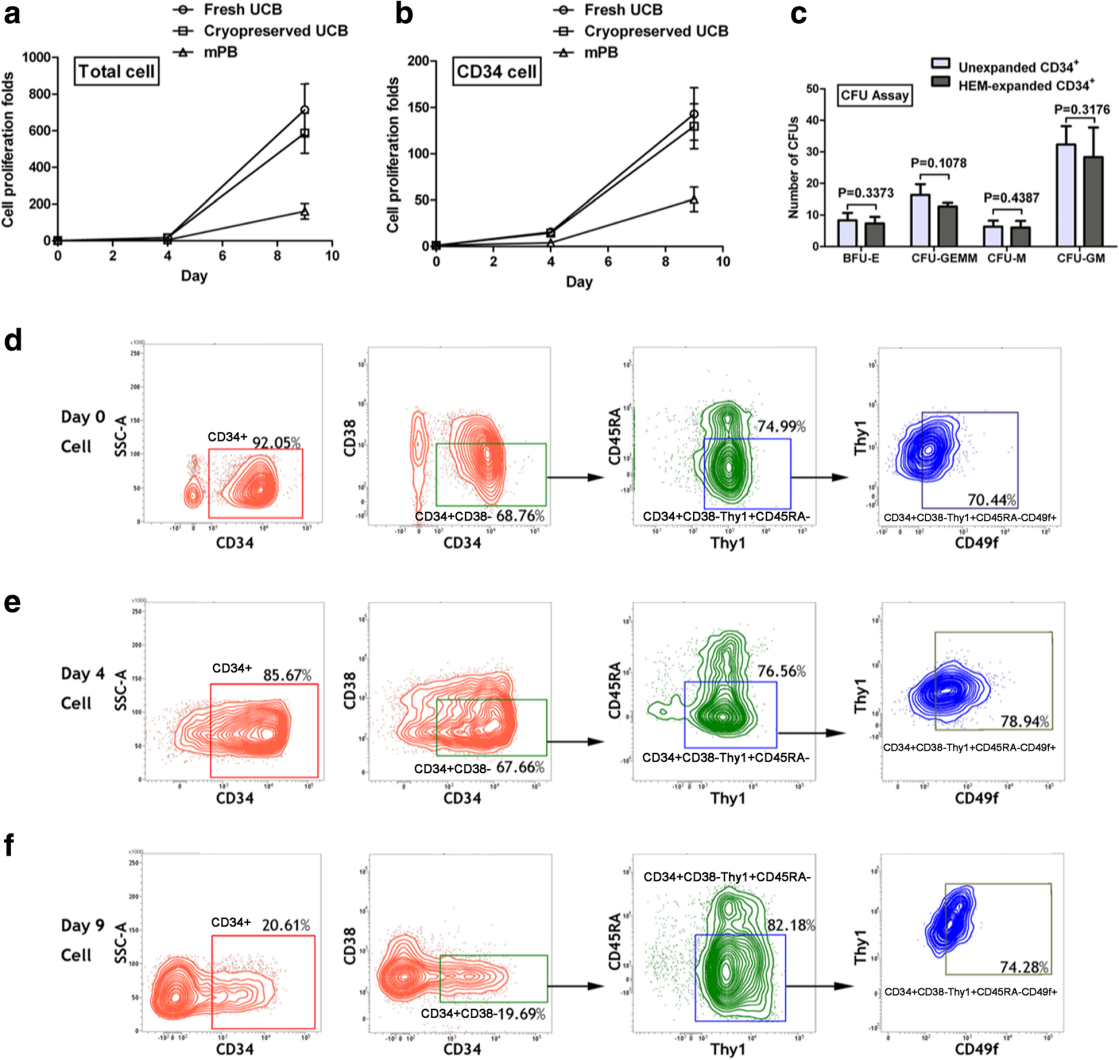
Effects of HEM on the ex vivo expansion of human HSCs from different sources. Human CD34^+^ cells purified from fresh UCB, cryopreserved CB, and mPB were cultured with HEM to day 4 or day 9. Total cell (**a**) and CD34^+^ cell (**b**) expansion was calculated as fold increase (after/before expansion) in cell counts on each day (days 0, 4, and 9). Data are shown as mean ± SD, *n* = 10. **c** Types of CFU colonies formed from the hematopoietic stem/progenitor cells before/after expansion. Equal numbers of unexpanded human CD34^+^ cells and 9-day HEM-expanded CD34^+^ cells (1 × 10^4^ per dish) were assayed as described in Methods. Data are shown as mean ± SD, *n* = 4. Pairwise comparisons (unexpanded vs expanded) were performed for each type of colony using Student’s *t* test (*p* > 0.05 for all pairs). **d**–**f** Representative flow analysis for multi-immunophenotyping of the expanded cells on day 0, day 4, and day9. Gates of CD34^+^ CD38^−^, CD90^+^, CD45RA^−^, and CD49f^+^ cell populations were set based on the fluorescence minus one (FMO) of each cell surface marker antibody. CD38^−^, CD90^+^, CD45RA^−^, and CD49f^+^ were analyzed in CD34^+^ cell population

We compared the ability of HEM to expand HSCs derived from fresh human UCB, cryopreserved human UCB, or human mPB. In general, during the first 4 days, the cells expanded at a slow pace, whereas from day 4 to day 9, cells cultured with HEM exhibited overall exponential growth (Fig. 2a), with rapid increases in both total and CD34^+^ cells (Fig. 2b). On day 4, the fold-changes in proliferation for CD34^+^ cells from fresh UCB, cryopreserved UCB, and cryopreserved mPB were 15.63 ± 1.76, 14.41 ± 2.72, and 3.85 ± 1.02, respectively. On day 9, the fold-changes were 142.9 ± 28.4, 129.7 ± 24.3, and 50.9 ± 13.2, respectively. These results demonstrated that HEM could promote the expansion of CD34^+^ cells from all three sources. There was no significant difference between the fresh and cryopreserved human UCB cultures with respect to rates of CD34^+^ proliferation, but CD34^+^ cells from UCB exhibited higher proliferation potential than those from mPB. Hence, subsequent experiments were performed with cryopreserved UCB samples.

### Immunophenotyping of expanded HSC

To further define whether the ex vivo expanded CD34^+^ cells possessed the other acknowledged HSC’s immunologic characteristics, including the presence of CD90 and absence of CD45RA [41], the percentage of CD34^+^ cells in the total cell population (P1) and the percentage of CD38^−^CD90^+^CD45RA^−^CD49f^+^ cells in the CD34^+^ cell group was measured (Fig. 2d–f). The proportion and absolute number of CD34^+^CD38^−^CD90^+^ CD45RA^−^CD49f^+^ cells were calculated and compared among each group (SGF 0–6) on day-0, day-4 and day-9 (Additional file 1: Table S1). It could be seen that the absolute number of CD34^+^CD38^−^CD90^+^CD45RA^−^CD49f^+^ cells in group SGF 6 significantly increased, compared to groups SGF 2–5 on both day 4 and day 9, indicating that HEM can support the expansion of CD34^+^CD38^−^CD90^+^CD45RA^−^CD49f^+^ cells. The above results indicated that although a part of initial CD34^+^ cells differentiated during the culture resulting in decreased CD34^+^ proportion, there still existed plenty CD34^+^ cells maintained self-renewal and proliferation, contributing to significant increase of CD34^+^CD38^−^CD90^+^CD45RA^−^CD49f^+^ cells. In addition, we isolated HEM-expanded CD34^+^ cells and performed CFU assay, and the results showed that HEM-expanded CD34^+^ cells were capable of forming various colonies including BFU-E, CFU-GM, CFU-GEMM, and CFU-M, further proving their ability to form different types of blood cells (Fig. 2c). The immunophenotyping of various blood cells in HEM-expanded cells were also detected on day 0, day 4, and day-9 (Additional file 1: Table S2).

### Engraftment of HEM-expanded HSCs in NOD/SCID mice

**Fig. 3:**
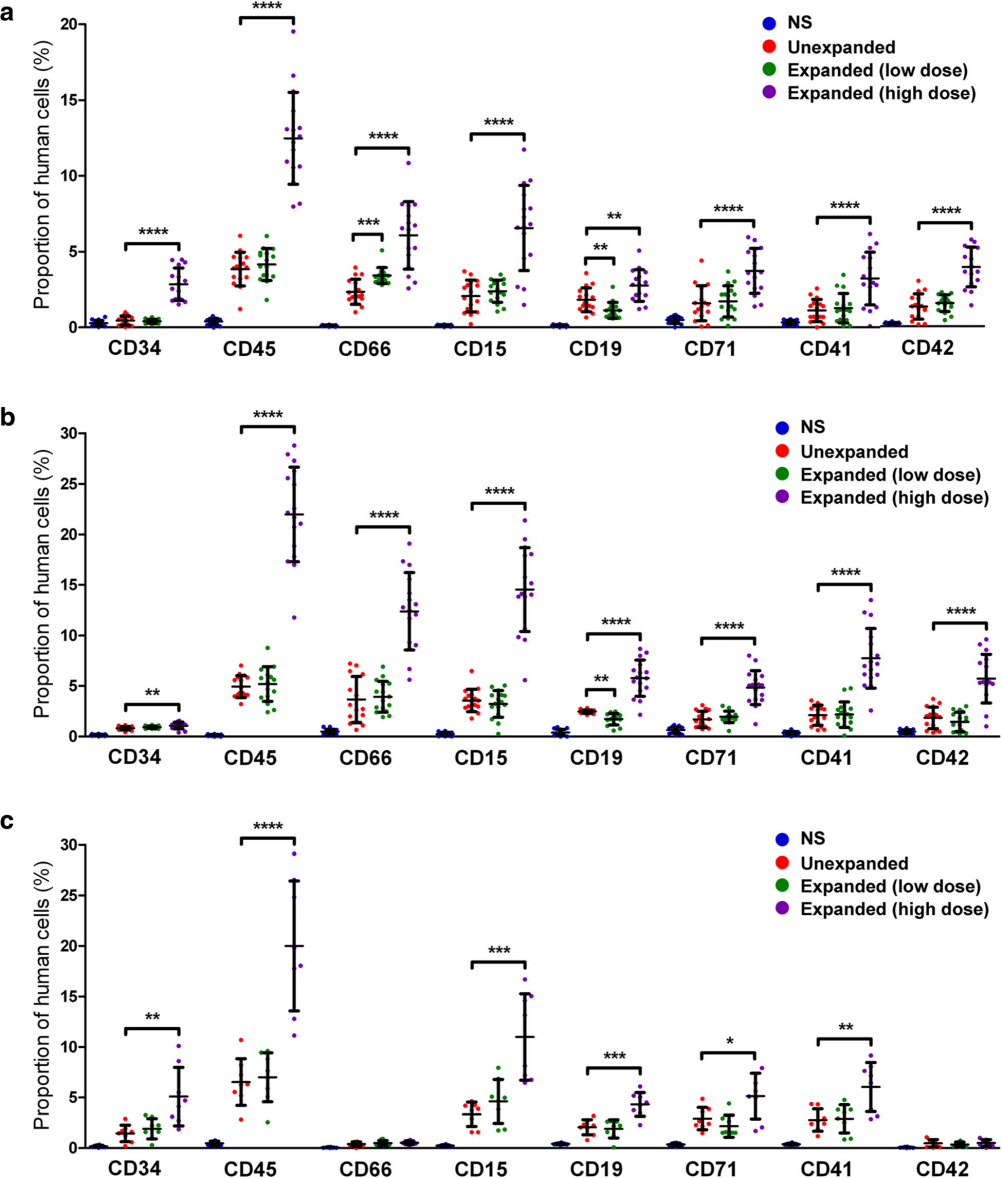
Functional assessment of human cells in PB and BM of NOD/SCID mice transplanted with various types of human HSCs. Three weeks (**a**) and eight weeks (**b**) after intravenous human CD34^+^ transplantation in mice, the presence of human cells was analyzed in the PB of mice transplanted with unexpanded human HSCs, low-dose expanded HSCs, or high-dose expanded HSCs. Normal saline (NS) was injected as the vehicle control. Data are shown as mean ± SD, *n* = 16. **c** At week 8 post-transplantation, engrafted human cells were detected in the BM by flow cytometry. The percentages of cells expressing human hematologic-lineage markers shown are calculated on the total (mouse plus human) cell population from mouse PB or BM. Data are shown as mean ± SD, * *p* < 0.05, **p < 0.01, ***p < 0.001, ****p < 0.0001; one-way ANOVA followed by Dunnett’s multiple comparison test

Given the lower immunogenicity and higher proliferation potential of UCB cells relative to blood cells from other sources, the ever-increasing feasibility of identifying HLA-matched UCB HSCs from many storage facilities worldwide, and the availability of UCB samples for the current study, we conducted the in vivo NOD/SCID transplantation studies using HEM-expanded human UCB CD34^+^ cells. Engraftment of human hematopoietic cells was determined using NOD/SCID mice subjected to sub-lethal irradiation (radioactive source ^60^Co; dose 2.5 Gy; radioactive intensity 0.38 Gy/min). Flow cytometric analysis of PB and BM cells revealed that human cell surface antigens were not expressed in cells from control mice, which were injected with saline. Three weeks after transplantation, human CD34^+^ cells were detected at 2.84 ± 1.03% in mouse PB in the high-dose group (transplanted with all the cells expanded from 1 × 10^5^ starting CD34^+^ cells), whereas the proportion was below 0.5% in both the unexpanded group (transplanted with 1 × 10^5^ unexpanded CD34^+^ cells), and the low-dose cell group (transplanted with 1 × 10^5^ ex vivo expanded CD34^+^ cells) (Fig. 3a). Cells from mouse PB were also positively stained for human CD45, CD66, CD15, CD19, CD71, CD41a, and CD42b in all groups that received HSCs, indicating the contribution of injected human HSCs to different types of blood cells. Relative abundance of CD34^+^, CD45^+^, CD15^+^, and CD19^+^ cells were significantly greater in the high-dose expanded group than in the other groups.

Eight weeks post-transplantation, human CD45^+^, CD66^+^, CD15^+^, CD19^+^, CD71^+^, CD41a^+^, and CD42b^+^ cells were easily detectable in mouse PB in all groups that received HSCs. The relative cell abundance in the unexpanded group and the low-dose expanded group was similar, whereas that of the high-dose expanded group was significantly greater than the other groups, for all subtypes, with the fraction of CD45^+^ cells reaching > 20% (Fig. 3b). Moreover, compared to week 3 (Fig. 3a), abundance of most subtypes of human hematopoietic cells in the high-dose expanded group was significantly greater at week 8 (Fig. 3b; *p* < 0.001 for CD34^+^, CD15^+^, CD45^+^; *p* < 0.01 for CD19^+^, CD41^+^, CD66^+^; *p* > 0.05 for CD42^+^, CD71^+^).

Further analysis of mouse BM cells at 8 weeks post-transplantation showed that CD34^+^ cells were at 1.43 ± 0.78% in the unexpanded group, 1.92 ± 0.93% in the low-dose cell group, and 5.05 ± 2.71% in the high-dose cell group (Fig. 3c). CD45 marker was positive in these three groups at 6.50 ± 2.13%, 6.98 ± 2.72%, and 19.9 ± 8.2%, respectively. Other subtypes of human hematopoietic cells were also detected with the high-dose group showing a significantly greater abundance than the other groups for CD15^+^ and CD19^+^ cells.

**Fig. 4:**
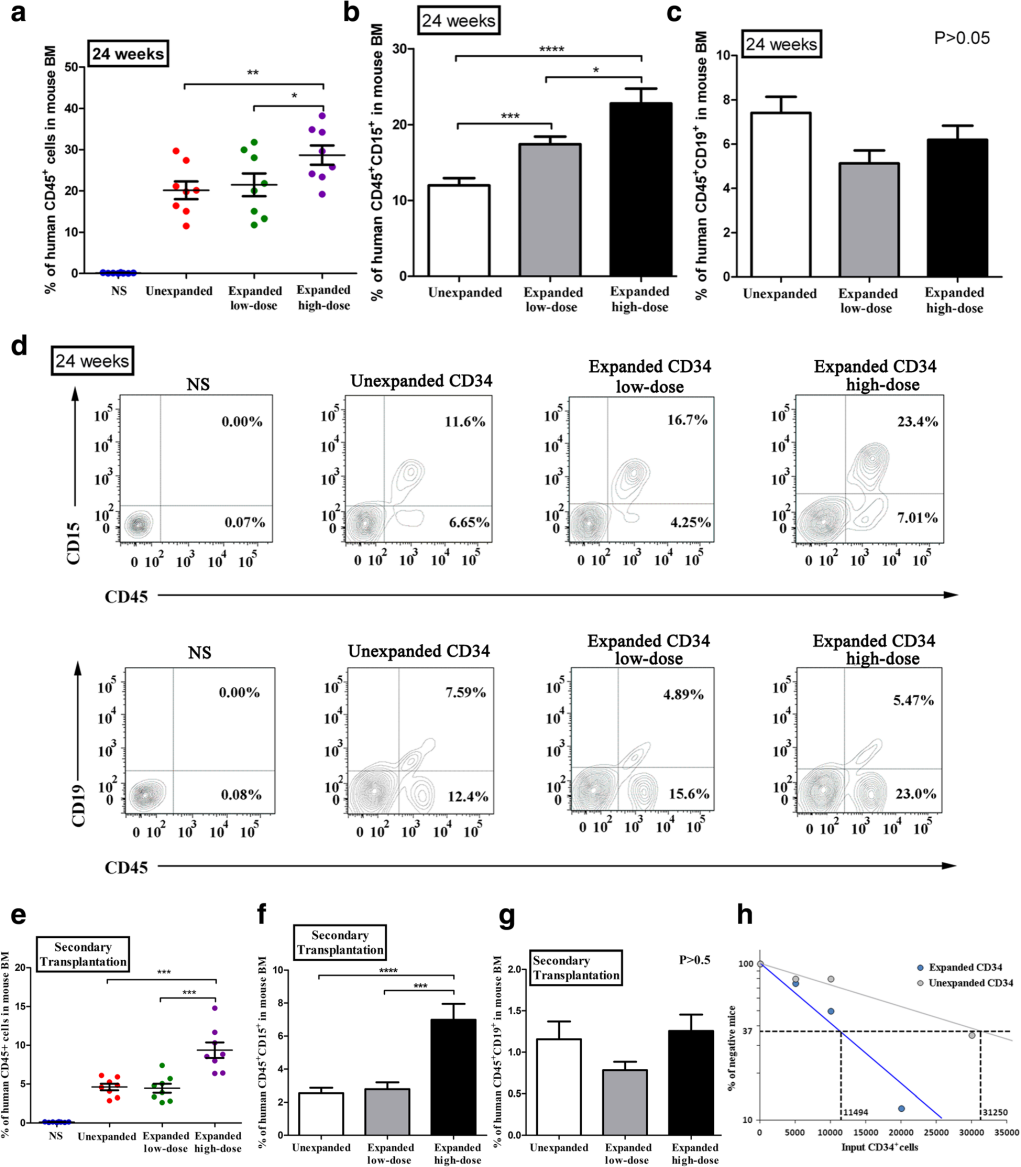
Long-term engraftment of human cells in NOD/SCID mice. **a** Extent of human cell engraftment in the BM of NOD/SCID mice transplanted with normal saline (NS; blue dots), unexpanded HSCs (red dots), low-dose expanded HSCs (green dots), and high-dose expanded HSCs (purple dots) at 24 weeks post-transplantation. **b**, **c** Proportion of CD45^+^CD15^+^ and CD45^+^CD19^+^ cells in the three HSC transplantation groups at week 24 post-transplantation. **d** Representative flow cytometry of human CD45^+^CD15^+^ (myeloid) lineage and CD45^+^CD19^+^ (lymphoid) lineage in mouse BM at 24 weeks post-transplantation. **e** Extent of human cell engraftment in mouse BM at 8 weeks following secondary transplantation in NOD/SCID mice. Data points are color-coded for each group as **a**. **f**, **g** Proportion of CD45^+^CD15^+^ and CD45^+^CD19^+^ cells in the three HSC transplantation groups at week 8 after secondary transplantation. Data are shown as mean ± SD, *n* = 8 in each group. * *p* < 0.05, **p < 0.01, ***p < 0.001, ****p < 0.0001; one-way ANOVA followed by Dunnett’s multiple comparison test. **h** Comparison of the frequency of SRC in unexpanded CD34^+^ cells and HEM-expanded CD34^+^ cells. The human CD45^+^ cell proportion in the mouse BMs was analyzed by flow cytometry 8 weeks after transplantation

Significantly, over 20% leukocytes isolated from mouse BM remained positive for human CD45 at week 24 after transplantation (Fig. 4a). Furthermore, the percentages of the specific types of human cells in the unexpanded group, low-dose expanded group, and high-dose expanded group were 12.0% ± 2.5%, 17.4% ± 2.7%, and 22.8% ± 5.2% (***p < 0.001 among three groups) for CD45^+^CD15^+^ myeloid cells (Fig. 4b, d), respectively, and 7.4% ± 1.9%, 5.1% ± 1.6%, and 6.2% ± 1.7% (*p* = 0.0684 among three groups, one-way ANOVA, no significant difference) for CD45^+^CD19^+^ lymphoid cells (Fig. 4c, d), respectively. These result indicated that, although there was a trend of increase in myeloid potential, the expansion procedure did not noticeably compromise the lymphoid potential for the engraftment.

To further validate the presence of long-term repopulating cells after ex vivo culture, secondary transplantation was conducted. At 8 weeks after secondary transplantation, flow cytometric analysis revealed that cells positive for human CD45^+^ were at 4.25 ± 1.60%, 4.13 ± 1.71%, and 8.99 ± 3.10% among total BM cells in the unexpanded group, the low-dose group, and high-dose group, respectively (Fig. 4e). In addition, these three groups of mice were all positive for human CD15^+^ (myeloid) and CD19^+^ (lymphoid) cells and that, while the abundance of the myeloid lineage appeared greater in the high-dose expanded group than in the other groups, the lymphoid potential for the engraftment was not compromised by the expansion procedure (Fig. 4f, g). Therefore, it can be concluded that human HSCs derived from ex vivo expansion with HEM contained a considerable number of long-term HSCs with the ability to repopulate in BM and differentiate into different types of blood cells.

Moreover, limiting dilution analysis was employed to clarify the frequency of SRCs in unexpanded CD34^+^ cells and HEM-expanded CD34^+^ cells. As shown in Fig. 4h, the SRC frequencies were estimated to be 1/31250 and 1/11494 in unexpanded CD34^+^ cells and HEM-expanded CD34^+^ cells, respectively. According to the result, SRC frequency was found to be enhanced by 2.7 fold in mice that received HEM-expanded cells as compared to those receiving unexpanded cells in week 8 post-transplantation. This data was consistent with the results of immunophenotyping of the HEM-expanded cells, which implied that long-term HSCs were enriched in the expanded HSC group and that the HEM platform could be used to effectively expand human HSCs.

### Expansion and transduction of mobilized primate PB-CD34^+^ cells

**Fig. 5:**
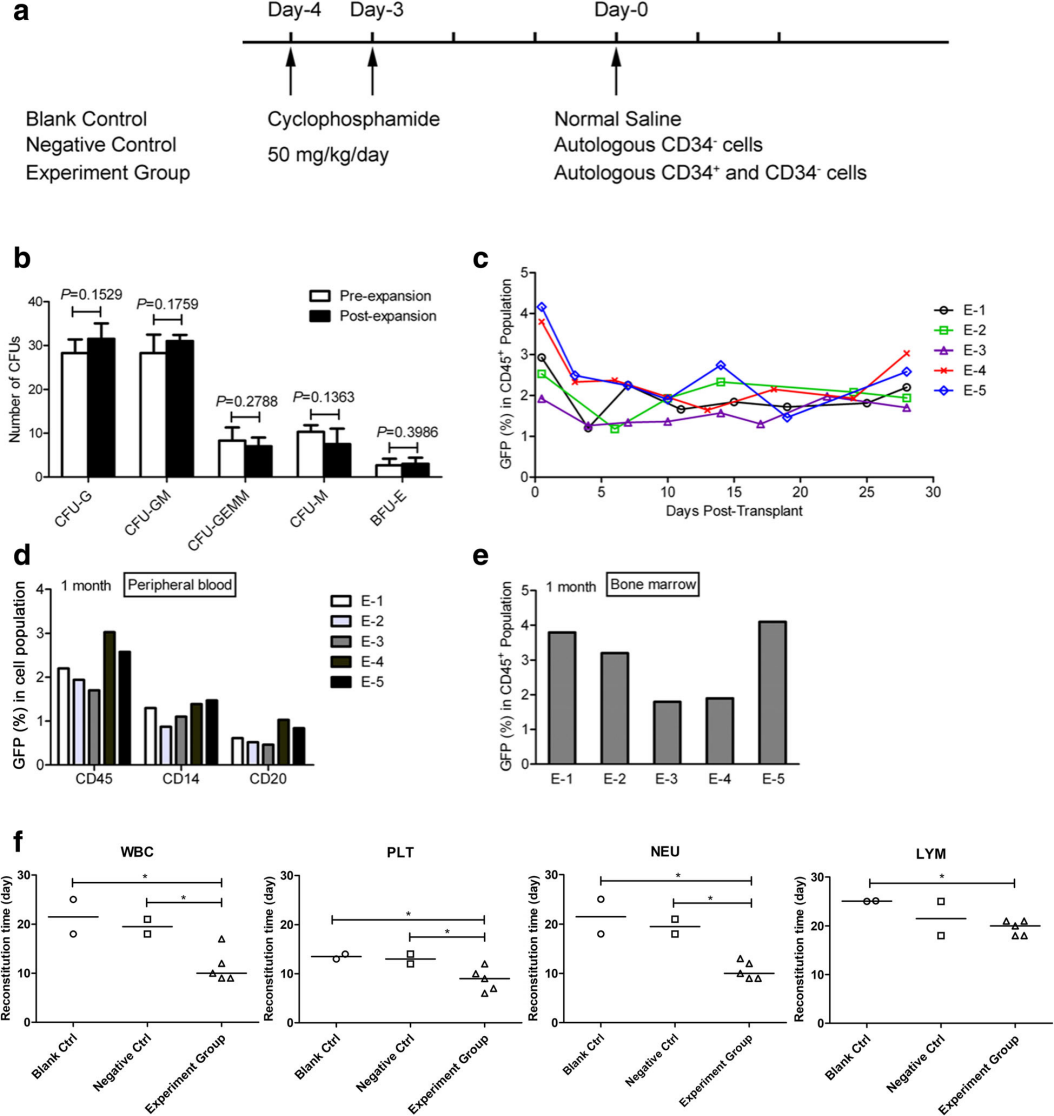
Autologus transplantation in a nonhuman primate model. **a** Scheme for autologus transplantation in a nonhuman primate model. CTX treatment was administered on days − 4 and − 3, and transplantation was performed on day 0. **b** Types of CFU colonies formed from fresh isolated CD34^+^ cells (pre-expansion) and 9-day HEM-expanded cells (Post-expansion). Data are shown as mean ± SD, *n* = 4. **c** Percentage of GFP^+^ cells in PB nucleated cells (CD45^+^ population) at various time points during the first month following autologous transplantation. **d** Percentage of GFP^+^ cells in primate PB CD45^+^, CD14^+^, and CD20^+^ cell population at 1 month post-transplantation. **e** GFP^+^ cells in the primate BM CD45^+^ population at 1 month after transplantation. PB and BM samples were from experimental group (E-1, E-2, E-3, E-4, and E-5). BM was harvested from primate femora. **f** Recovery time of white blood cell (WBC), neutrophil (NEU), platelet (PLT), and lymphocyte (LYM) were determined by comparing the baseline before/after CD34^+^ cell transplantation. Blank control, *n* = 2; negative control, *n* = 2; experiment group, *n* = 5. The lines of each group indicate the median in statistical analysis

To further and better evaluate the safety and efficacy of our expansion strategy HEM, an in vivo study in the primate model was performed as part of the preclinical evaluation (Fig. 5a). Given that no immunosuppressed primate models are available to test the function of ex vivo expanded human CD34^+^ cells, and the difficulty in obtaining primate UCB, we use autologous primate mPB CD34^+^ cells for transplantation. CD34^+^ cells from nonhuman primates were purified from mPB and then cultured ex vivo in HEM. After a 6-day expansion period, the cells were transduced with a GFP lentivirus and re-expanded for three additional days. Over the 9-day culture, total nucleated cells and CD34^+^ cells expanded significantly with an average of 63.7% (ranging from 55.9 to 71.7%) of CD34^+^ cells. Compared with the unexpanded CD34^+^ cells, expanded PB CD34^+^ cells from the primates exhibited no significant loss in typical CD34 expression during ex vivo culture. The fold-change in CD34^+^ cell expansion ranged from 6.3 to 9.8 as indicated in Table 2. HEM-expanded primate CD34^+^ cells retained their hematologic-lineage differentiation ability based on the data from CFU assays (Fig. 5b).

**Table 2:** Summary of relevant information of the cynomolgus primates before/after transplantation

Items		B-1	B-2	B-3	N-1	N-2	N-3	E-1	E-2	E-3	E-4	E-5	Median^a^
Age (year)		7	7	7	6	7	7	6	7	7	7	6	7
Sex		Male	Male	Male	Male	Male	Male	Male	Male	Male	Male	Male	NA
Weight (kg)		6.5	5.6	8.0	6.2	6.4	6.1	5.8	4.6	6.6	6.6	6.6	6.4
Before expansion	Cell number	–	–	–	–	–	–	3.0× 10^6^	2.6× 10^6^	3.6× 10^6^	2.7× 10^6^	2.4× 10^6^	2.7× 10^6^
	CD34^+^ cell purity (%)	–	–	–	–	–	–	76.0%	64.9%	61.5%	73.6%	72.1%	72.1%
After expansion	Total cell number	–	–	–	–	–	–	2.0 × 10^7^	1.7 × 10^7^	2.3 × 10^7^	2.3 × 10^7^	2.2 × 10^7^	2.2 × 10^7^
	CD34^+^ cell purity (%)	–	–	–	–	–	–	71.7%	55.9%	57.1%	68.3%	65.6%	65.6%
Expansion fold	Total cell	–	–	–	–	–	–	8.0	7.4	6.8	10.0	10.8	8.0
	CD34^+^ cell	–	–	–	–	–	–	7.5	6.4	6.3	9.3	9.8	7.5
Transduction efficiency (GFP %)		–	–	–	–	–	–	31.4%	37.1%	32.8%	31.2%	40.4%	32.5%
Transplant dose (per kg)	CD34^+^ cell	–	–	–	–	–	–	2.4 × 10^6^	2.0 × 10^6^	2.0 × 10^6^	2.4 × 10^6^	2.2 × 10^6^	2.2 × 10^6^
	CD34^−^ cell	–	–	–	2.6 × 10^7^	2.0 × 10^7^	2.5 × 10^7^	2.6 × 10^7^	2.5 × 10^7^	2.0 × 10^7^	2.5 × 10^7^	2.5 × 10^7^	2.5 × 10^7^
Outcome		Alive	Alive	Died	Alive	Alive	Died	Alive	Alive	Alive	Alive	Alive	NA

B-1, B-2, and B-3 were three animals in blank control; N-1, N-2, and N-3 were three animals in negative control; E-1~E-5 were five animals in experiment group

^a^The median was calculated of the experiment group

*Abbreviations*: *NA* not applicable, *GFP* green fluorescent protein

### Hematological recovery of primates after autologous transplantation

A nonhuman primate myelo-suppression model was established for autologous transplantation by CTX treatment, resulting in remarkably declined whole blood cell counts. CD34^+^ cells (ranging from 2.0 ×  10^6^ to 2.4 × 10^6^/kg), and CD34^−^ cells (ranging from 2.0 × 10^7^ to 2.6 × 10^7^/kg) were used for autologous transplantation in primates (Table 2). Time-dependent recovery of WBC (Additional file 1: Figure S1), PLT (Additional file 1: Figure S2), NEU (Additional file 1: Figure S3), and LYM (Additional file 1: Figure S4) was recorded in the group transplanted with CD34^+^ cells. In the autologous CD34^+^ transplantation group, the WBC, NEU count nadir after transplantation declined to approximately 25% or < 25% of the initial value whereas in the control groups, the count nadir was 10% or < 10% of the initial value. A range of 50 to 60% of the initial PLT count nadir was observed in autologous CD34^+^ transplantation, whereas it was 25~35% in both control groups. In the autologous CD34^+^ transplantation group as well as in the control groups, the LYM count nadir after transplantation was about 10% or < 10% of the initial value. These results showed that transplantation with expanded CD34^+^ cells could improve the NEU and PLT count nadir, which is helpful for hematological recovery.

As shown in Fig. 5f, WBC, NEU, and PLT in primate PB were restored to their normal levels on days 10, 10, and 9 after autologous transplantation with expanded CD34^+^ cells, respectively. Restoration of WBC, NEU, and PLT to the normal level occurred on days 19.5, 19.5, and 13, respectively, in the CD34^−^ control group and on days 21.5, 21.5, and 13.5 days, respectively, in the saline control group. However, lymphocytes were completely restored on day 20 post autologous CD34^+^ cell transplantation, compared to in the CD34^−^ group (day 21.5) or the saline group (day 25). These data demonstrated that the expanded cells were able to achieve superior recovery of NEU and PLT in a timely manner following the myelo-suppressed condition. During the first month post autologous transplantation, the ratio of GFP^+^ cells in the CD45^+^ population was around 1~3% in primate PB (Fig. 5c). GFP^+^ cells have a higher percentage in the CD14^+^ subset than that in the CD20^+^ subset (Fig. 5d), suggesting that the expanded CD34^+^ cells had excellent properties for accelerating hematopoietic recovery in primates, especially for the myeloid lineage. One month after transplantation, GFP^+^CD45^+^ cells ranging from 1.8 to 4.1% were also detectable in primate BM (Fig. 5e), indicating that the expanded CD34^+^ cells were capable of engraftment into primate BM.

### Long-term survival of primates transplanted with expanded autologous CD34^+^ cells/progenitors

Two myelo-suppressed primates (one from the negative control group and the other from the autologous CD34^−^ cell group) survived only 5 days after transplantation. Both animals died of pancytopenia and acute renal failure. BM biopsy was performed at the time of euthanasia, which confirmed a dramatic decrease in nucleated BM cells. These observations indicated that these primates could not withstand the CTX-induced myelo-suppression. Contrarily, five cynomolgus primates that received the expanded CD34^+^ cells were all apparently normal during and after the course of experiment. By 18 months post-transplantation, these primates continued to survive with no apparent abnormalities with regard to parameters including complete blood counts, blood chemistries, appetite, weight gain, behavior, and social interaction. These results implied that transplantation of expanded cells resulted in no adverse reactions in primates.

## Discussion

In this study, a medium formulation (HEM) of the cytokine cocktail in IMDM combined with nutritional supplements (selenium, putrescine, transferrin, insulin, B-27 supplements, and human serum albumin) has been successfully developed for efficient expansion of HSCs ex vivo, while retaining their hematologic-lineage differentiation, long-term repopulation, and hematopoietic recovery ability. Notably, this protocol does not require the use of stromal cells, animal components, or gene manipulation, valuable for industrialization and clinical applications, both in efficiency and in cost.

Using HEM, we have achieved excellent expansion of cryopreserved human UCB CD34^+^ cells with up to 129-fold increase and 2.7-fold increase in SRC frequency after 9 days of culture. Furthermore, since only CD34 marker alone cannot represent actual HSCs, we measure CD38^−^CD90^+^CD45RA^−^CD49f^+^ population which has been proved to represent HSCs that are capable of long-term engraftment after transplantation [41]. The results show that the proportion of CD38^−^CD90^+^CD45RA^−^CD49f^+^ cells in the CD34^+^ cell population is maintained and the absolute number of CD34^+^CD38^−^CD90^+^CD45RA^−^CD49f^+^ cells increase up to 190-fold compared with unexpanded CD34^+^ cells (Fig. 2d, e, Additional file 1: Table S1).

Cytokines and other growth factors play a vital role in any HSC expansion system [42–45]. In previously reported studies, some liquid culture systems could expand human HSCs by ~ 100-fold [6, 46–49], but at least 2 or 3 weeks ex vivo were needed. Ueda and coworkers [50] reported that the cytokine combination consisting of SCF, FL, TPO, IL-6, and soluble IL-6 receptor in a medium supplemented with 20% FBS and two other compounds achieved a 4.2-fold increase in SRC frequency. However, this condition seemed not optimal, given the addition of FBS and the low expansion folds (total cell ~ 45.4-fold and CD34^+^ cell ~ 6.1-fold). Amsellem and coworkers [51] provided an approach where the HSCs were cultured on stromal cells genetically engineered to secrete HOXB4, resulting in expansions of total cells by ~ 530-fold, and CD34^+^ cells by ~ 122-fold, within 4–5 weeks and increased in SRC frequency by 2.5-fold. However, it is well known that the introduction of stromal cells may cause immunological effects in clinical transplantation. Considering potentially negative clinical effects, stromal cells, animal components, or gene manipulation should be avoided in HSC culture procedure. Thus, compared with above published approaches, our optimized procedure has advantages in the relatively high CD34^+^ expansion fold, the maintenance of long-term repopulating and hematologic-lineage differentiation ability, and the production of clinical-leveled pilot scale using a turning-bottle device. Besides, it is well known that one of the most important deciding factors for any cell source and any protocol is the associated costs. The cost of HEM is estimated as only 1/20 compared with the mostly common commercialized HSC expansion medium. Thus, the HEM formula that we have developed for human HSC expansion is efficient and cost effective.

Human long-term HSCs are usually determined by evaluating the ability of long-term repopulation in xenograft models—specifically, the total engraftment time of primary and secondary transplants should be more than 8 weeks [52]. Therefore, we have performed primary transplantation for up to 24 weeks and secondary transplantation for 8 weeks in NOD/SCID mice. The results show that mice transplanted with unexpanded HSCs and a low dose of expanded HSCs have similar proportions of engraftment, and mice infused with a high dose of expanded HSCs contain even more human cells, indicating that HEM-expanded human HSCs retain BM engraftment and long-term repopulation ability in vivo. Furthermore, the result of limit dilution assays confirms the frequency of long-term HSCs in HEM-expanded CD34^+^ cells persuasively (Fig. 4a–d).

Notably, we observed that upon 24-week engraftment in NOD/SCID mice, the percentage of CD45^+^CD15^+^ (myeloid) population was higher in the expanded groups than in the unexpanded group, whereas that of the CD45^+^CD19^+^ (lymphoid) population was similar among all three groups. This result indicates that, although the HEM-expanded HSCs have a tendency to preferentially reconstitute the myeloid lineage, their potential to reconstitute the lymphoid lineage is as robust as unexpanded cells. This notion is confirmed by results from the secondary transplantation experiment, where the abundance of the lymphoid population is also similar among all three groups.

Although we have verified the homing and long-term engraftment ability of HEM-expanded HSCs in the NOD/SCID mouse model, it is still necessary to evaluate their ability to promote hematological recovery. Because of xenotransplantation, it is difficult to assess the mouse hematopoietic recovery by transplanting human HSCs. Therefore, we performed autologous transplantation of primate HEM-expanded CD34^+^ cells in a myelo-suppressed primate model. The myelo-suppressed primate model has been developed by CTX treatment, leading to a sharp decline in multiple blood cell lineages. Compared to the myelo-suppressed primate models generated by whole body radiation, CTX-treated primates are more convenient for preclinical studies to evaluate the function of expanded cells. To our knowledge, this is the first study of transplantation of CD34^+^ cells in a CTX-induced myelo-suppressed nonhuman primate model. It is valuable for providing a new platform to evaluate the functions of hematopoietic cells induced by cytokines, small chemical compounds, and/or any other ex vivo stimulating systems prior to clinical application.

Using the same culture medium, the expansion efficiency of CD34^+^ cells derived from primate mPB was relatively lower than that obtained from humans. The possible explanation for this observation may be that the cytokines used are all recombinant human proteins, which results in reduced activities on primate cells. In addition, culture conditions for primate cells are not optimized because our goal is only to validate the safety and efficacy of HEM-expanded CD34^+^ cells in primates. Nevertheless, our findings demonstrate that nonhuman primate CD34^+^ cells could also be expanded to the level of clinical significance.

The results of primate autologous transplantation have proved that HEM-expanded CD34^+^ cells can shorten the period of pancytopenia and enhance the hematological recovery after myelo-suppression. Primates transplanted with the expanded CD34^+^ cells exhibited superior hematopoietic recovery compared to control groups and showed no adverse effects as long as 18 months after transplantation, whereas both control groups (CD34- or saline) showed death on day 5 post-transplantation. Moreover, the expanded CD34^+^ cells could increase the count nadir of NEU and PLT and reduce the susceptibility to infection following transplantation, thus enhancing survival. In addition, NEU, WBC, and PLT from primates transplanted with the expanded CD34^+^ cells were restored to normal levels (approximately within 1.5–2 weeks) faster than LYM (3 weeks), and the median time of LYM recovery did not show a significant difference compared with the control groups. Furthermore, at 1 month post-transplantation, the subset staining results from primate PB demonstrated that GFP^+^ cells were higher in CD14^+^ (myeloid) population than in the CD20^+^ (lymphoid) population. Coincidentally, we also observed that upon secondary transplantation in NOD/SCID mice, the percentage of CD45^+^/CD15^+^ (myeloid) population was higher than that of the CD45^+^/CD19^+^ (lymphoid) population. All these results indicate that HEM-expanded CD34^+^ cells preferentially reconstitute the myeloid lineage.

In primates transplanted with GFP-labeled cells, the percentage of GFP^+^ cells was relative low and they could not be detectable by 3~6 months post-transplantation. In terms of the low GFP percentage and short detection window, there may be two reasons: (1) the transduction efficiency of GFP lentiviral particles for expanded primate cells is only between 30 and 40% (Table 2), which may cause the detected GFP^+^ cells to be less than totally engrafted CD34^+^ cells; (2) there are still some residual primate bone marrow cells after myelo-suppression by CTX, and these cells could be recovered over time. As a result, exogenously labeled GFP^+^ cells could be diluted as the residual primate HSCs gradually recover.

Successful transplantation with autologous CD34^+^ cells in the nonhuman primate model strongly suggests a possible application for treating certain blood diseases. The effectiveness of the HEM expansion strategy applies not only to UCB cells, but also to HSCs derived from mPB, where approximately 50 CD34^+^ cells could be obtained via ex vivo expansion from a single human mPB-derived CD34^+^ cell. We propose that personalized, autologous transplantation can be developed for certain blood-related diseases, such as leukemia, using normal CD34^+^ cells isolated from a small volume of mPB after efficient ex vivo expansion in HEM. This strategy would minimize the suffering of patients due to the use of blood cells isolated through conventional approaches. Furthermore, given that there are many state-of-the-art facilities that store human UCB HSCs worldwide, it is also convenient to search for HLA-matched UCB HSCs. The amount of expanded HSCs derived from one CB unit (including those with lower CD34 content) was, in theory, still sufficient for adult (with an average weight of 70 kg) transplantation to treat malignant blood disorders.

## Conclusions

In conclusion, we have developed a new, low-cost medium formulation (HEM) that can greatly enhance the expansion of HSCs while retaining their long-term repopulation, hematologic-lineage differentiation and hematopoietic recovery ability. Moreover, autologous transplantation studies in nonhuman primates strongly suggest that HEM-expanded CD34^+^ cells could be safe and efficacious in potential clinical applications.

## Additional file

Additional file 1: **Table S1.** Proportion and absolute number of CD34^+^CD38^-^CD90^+^CD45RA^-^CD49f^+^cell^*a*^. **Table S2.** Immunophenotyping oftotal cells derived from ex vivo expansion of human UCB CD34^+^Cells in HEM^*a*^. **Figure S1.** Recovery of white blood cell(WBC)in primates over the course of autologous transplantation. **Figure S2**. Recovery of platelet (PLT)in primates over the course of autologous transplantation. **Figure S3.** Recovery ofneutrophil (NEU) in primates over the course of autologous transplantation. **Figure S4.** Recovery of lymphocyte (LYM) in primates over the course of autologous transplantation. (DOCX 274 kb)

## Abbreviations

3 Rs: Regulations of replacement refinement and reduction
APC: Allophycocyanin
BFU-E: Erythroid burst-forming unit
BM: Bone marrow
CFU: Colony-forming unit
CFU-G: Granulocyte colony-forming unit
CFU-GEMM: Granulocyte-erythrocyte- macrophage-megakaryocyte colony-forming unit
CFU-GM: Granulocyte-monocyte colony-forming unit
CFU-M: Megakaryocyte colony-forming unit
CO_2_: Carbon dioxide
FITC: Fluorescein isothiocyanate
Flt-3 L: Fms-related tyrosine kinase 3 ligand
GFP: Green fluorescent protein
HoxB4: Homeobox B4
HSCs: Hematopoietic stem cells
MACS: Magnetic-activated cell sorting
MNCs: Mononuclear cells
mPB: Mobilized peripheral blood
NOD/SCID: Non-obese diabetic/severe combined immunodeficiency
PB: Peripheral blood
PE: Phycoerythrin
RT: Room temperature
SCF: Stem cell factor
SRC: SCID-repopulating cells
TPO: Thrombopoietin
UCB: Umbilical cord blood
