# Supplementary material for: Safety and efficacy of ex vivo expanded CD34+ stem cells in murine and primate models
Source: Stem Cell Res Ther. 2019 Jun 13;10:173. doi: 10.1186/s13287-019-1275-0 (PMC6567473; doi:10.1186/s13287-019-1275-0)
Supplement: Supplementary file 1 — Table S1. Proportion and absolute number of CD34+CD38-CD90+CD45RA-CD49f+cella. Table S2. Immunophenotyping oftotal cells derived from ex vivo expansion of human UCB CD34+Cells in HEMa. Figure S1. Recovery of white blood cell(WBC)in primates over the course of autologous transplantation. Figure S2. Recovery of platelet (PLT)in primates over the course of autologous transplantation. Figure S3. Recovery ofneutrophil (NEU) in primates over the course of autologous transplantation. Figure S4. Recovery of lymphocyte (LYM) in primates over the course of autologous transplantation. (DOCX 274 kb) [file 13287_2019_1275_MOESM1_ESM.docx]

**Table S1. Proportion and absolute number of CD34^+^CD38^-^CD90^+^CD45RA^-^CD49f^+^ cell*^a^***

| Group | Subgroup | Proportion (CD34^+^CD38^-^CD90^+^CD45RA^-^CD49f^+^/CD34^+^) | Absolute number  (CD34^+^CD38^-^CD90^+^CD45RA^-^CD49f^+^)*^b^* |
| --- | --- | --- | --- |
| Unexpanded cell |  | 33.11% ± 9.58% | 0.33 ± 0.10 |
| 4-day expanded cell | GF0*^c^* | - | - |
|  | SGF0*^c^* | - | - |
|  | SGF2 | 58.10% ± 9.82% | 1.08 ± 0.19 |
|  | SGF3 | 57.25% ± 9.38% | 2.80 ± 0.48  **** |
|  | SGF4 | 51.16% ± 8.19% | 3.79 ± 0.52  ****  *** |
|  | SGF5 | 48.32% ± 7.91% | 5.78 ± 1.26 |
|  | SGF6 | 45.47% ± 4.33% | 6.64 ± 1.75 |
| 9-day expanded cell | GF0*^c^* | - | - |
|  | SGF0*^c^* | - | - |
|  | SGF2 | 45.51% ± 7.32% | 5.99 ± 1.32 |
|  | SGF3 | 56.87% ± 10.23% | 12.30 ± 2.73  **** |
|  | SGF4 | 52.60% ± 8.15% | 24.63 ± 2.52  ****  **** |
|  | SGF5 | 46.57% ± 9.31% | 32.53 ± 2.96  **** |
|  | SGF6 | 50.62% ± 11.22% | 63.66 ± 8.87 |

*^a^*CD34^+^ cells and CD34^+^CD38^-^CD90^+^CD45RA^-^CD49f^+^ cells were detected by flow cytometry as described in Methods. *^b^*Absolute number of the CD34^+^CD38^-^CD90^+^CD45RA^-^CD49f^+^ cells which come from one single unexpanded CD34^+^ cell. The real absolute number of each group should be multiplied with 50000, the cell number seeded on day 0. *^c^*Rare cell survived in these groups. Results are presented as means ± SD of 4 independent experiments. ***, ****, P<0.001, P<0.0001, respectively, compared to SGF6 group. One-way ANOVA followed by Dunnett’s multiple comparison test.

**Table S2.** **Immunophenotyping of total cells derived from *ex vivo* expansion of human UCB CD34^+^ Cells in HEM*^a^***

| CD marker | Percentage of all cells | | |
| --- | --- | --- | --- |
|  | Day 0 (%) | Day 4 (%) | Day 9 (%) |
| CD45 | 97.99 ± 1.88 | 96.28 ± 3.09 | 81.67 ± 8.43 *** |
| CD34 | 95.35 ± 5.12 | 82.03 ± 11.07 | 31.49 ± 6.84 **** |
| CD34^+^ Thy1^+^CD45RA^-^ | 72.50 ± 5.05 | 58.60 ± 5.28 **** | 16.15 ± 1.83 **** |
| CD34^+^CD38^-^Thy1^+^CD45RA^-^CD49f^+^ | 31.11 ± 8.94 | 39.55 ± 2.69 * | 9.44 ± 2.00 *** |
| CD15 | 2.13 ± 1.09 | 4.09 ± 3.17 | 29.76 ± 7.32 **** |
| CD14 | 0.82 ± 0.15 | 1.37 ± 1.21 | 7.29 ±4.02 **** |
| CD19 | 0.13 ± 0.05 | 0.21 ± 0.17 | 0.11 ± 0.06 |
| CD3 | 0.05 ± 0.02 | 0.12 ± 0.07 | 0.11 ± 0.05 |
| CD71 | 1.85 ± 0.64 | 8.27 ± 5.83 ** | 5.52 ± 3.24 ** |
| CD41 | 2.17 ± 0.99 | 3.75 ± 2.63 | 9.94 ± 6.45 ** |

*^a^*Human UCB CD34^+^ cells were cultured in HEM for 0 (control), 4, or 9 days and the total cells were analyzed by flow cytometry for immunophenotyping, as described in Methods. Results are presented as means ± SD of 4 independent experiments. *, **, ***, ****, P<0.05, P<0.01, P<0.001, P<0.0001 respectively, compared to Day 0. One-way ANOVA followed by Dunnett’s multiple comparison test.

**Figure S1**





**Figure S1. Recovery of white blood cell** **(WBC) in primates over the course of autologous transplantation.** The WBC count on day -4 before cyclophosphamide treatment was regarded as the baseline. Recovery ratio of primates which received normal saline (A) (blank control) or CD34^-^ cells (B) (negative control) or CD34^+^ and CD34^-^ cells (C) (experiment group), were monitored following transplantation. Blank control, n=2; negative control, n=2; experiment group, n=5. The lines of each group indicate the median in statistical analysis.

**Figure S2**

**

**

**Figure S2. Recovery of platelet (PLT) in primates over the course of autologous transplantation.** The PLT count on day -4 before cyclophosphamide treatment was regarded as the baseline. Recovery ratio of primates which received normal saline (blank control) or CD34- cells (negative control) or CD34^+^ and CD34^-^ cells (experiment group), were monitored following transplantation. Blank control, n=2; negative control, n=2; experiment group, n=5. The lines of each group indicate the median in statistical analysis.

**Figure S3**





**Figure S3. Recovery of neutrophil (NEU) in primates over the course of autologous transplantation.** The NEU count on day -4 before cyclophosphamide treatment was regarded as the baseline. Recovery ratio of primates which received normal saline (blank control) or CD34^-^ cells (negative control) or CD34^+^ and CD34^-^ cells (experiment group), were monitored following transplantation. Blank control, n=2; negative control, n=2; experiment group, n=5. The lines of each group indicate the median in statistical analysis.

**Figure S4**

**

**

**Figure S4. Recovery of lymphocyte (LYM) in primates over the course of autologous transplantation.** The LYM count on day -4 before cyclophosphamide treatment was regarded as the baseline. Recovery ratio of primates which received normal saline (blank control) or CD34- cells (negative control) or CD34^+^ and CD34^-^ cells (experiment group), were monitored following transplantation. Blank control, n=2; negative control, n=2; experiment group, n=5. The lines of each group indicate the median in statistical analysis.
